# Combined Posterior-Anterior Interbody Fusion in the Management of Traumatic Lumbosacral Dissociation: A Case Report and Review of Literature

**DOI:** 10.7759/cureus.7089

**Published:** 2020-02-24

**Authors:** Kyle W Scott, Jonathan Arias, Kourosh Tavanaiepour, Daryoush Tavanaiepour, Gazanfar Rahmathulla

**Affiliations:** 1 Neurosurgery, University of Florida College of Medicine, Gainesville, USA; 2 Medicine, University of Florida College of Medicine, Gainesville, USA; 3 Neurosurgery, University of Florida College of Medicine, Jacksonville, USA; 4 Neurosurgery, University of Florida Health, Jacksonville, USA

**Keywords:** lumbosacral dissociation, short segment fusion, trauma, anterior fusion, posterior fusion

## Abstract

Traumatic lumbosacral dissociation is a unique, but well-documented, phenomenon that generally stems from high-energy impact injuries to the lower lumbar spine. Patients typically present with complicated and multisystem injuries with wide-ranging neurological deficits below the level of trauma. This presents stark challenges regarding the diagnosis, management, and surgical correction technique utilized. In this study, we present the case of a 21-year-old, morbidly obese, male patient that presented after a traumatic motor vehicle accident with L5-S1 lumbosacroiliac dissociation, cauda equina syndrome, and left lower extremity monoplegia. The degree of disruption warranted a 360° approach, we opted for an anterior lumbar interbody fusion followed by a posterior, lumbar interbody, short segment fusion. We review the case and relevant literature of similar lumbosacral dissociation studies with their management options and outcomes. Due to the rare nature of these devastating injuries, there remains wide variability in their management, with a combination of open anterior and posterior approaches resulting in variable long-term outcomes. The management of these rare injuries will require appropriate consideration of the patient’s unique etiology, coexisting injuries, and radiological imaging in deciding surgical stabilization techniques.

## Introduction

Traumatic lumbosacral dissociation (TLSD) is a rare injury that entails high-energy trauma to the spine or pelvis, typically resulting in multiple concomitant systemic injuries. Although an infrequent etiology, traumatic fracture-dislocation of the lumbosacral junction is a well-documented phenomenon. Most published reports and clinical series have shown that these injuries are commonly associated with motor vehicle accidents and are more prevalent in men than women, with an average age of around 27 years and typically bilateral dissociations with significant neurological deficits upon admission. The classification of complex lumbosacral injuries remains a controversial topic with various scoring systems taking into account the injury's morphology, posterior ligamentous complex integrity, and the patient's neurological status [1-3]. The severity of the injury results in chronic impairments, with early surgical intervention offering significant improvements in early mobilization, reduction in joint pain, mechanical stability, and possibly improved neurological outcomes [4-5].

## Case presentation

A morbidly obese (body mass index (BMI) = 47.57) 21-year-old male presented to our emergency department after being involved in a severe single motor vehicle, high-velocity accident. Emergency medical services (EMS) reported him as an unrestrained driver traveling at approximately 80 miles per hour with a collision resulting in a front-end rollover of his van. He was ejected out of his vehicle and found beneath it. His initial Glasgow Coma Scale (GCS) was 15 in the field, and he was in hemorrhagic shock.

**Figure 1:**
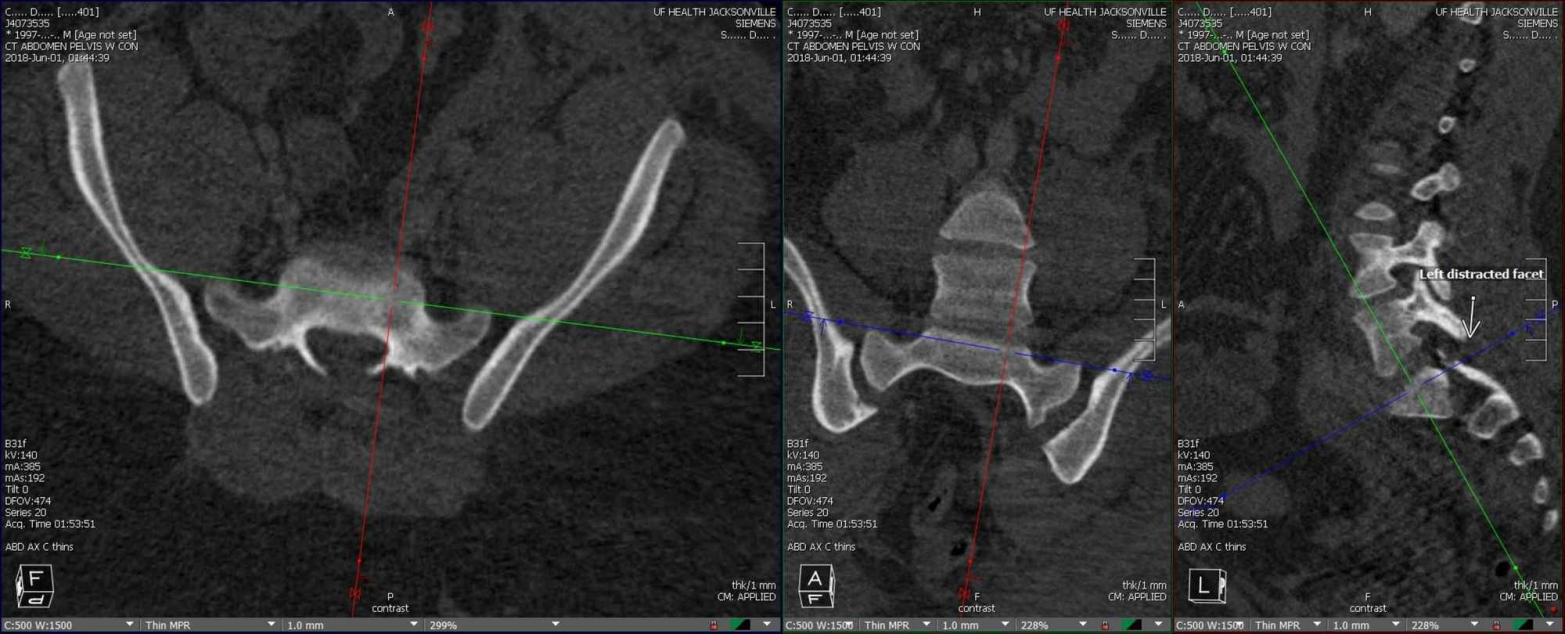
Computed tomography (CT) revealing traumatic lumbosacral dissociation Axial (left) and coronal (center) CT sections revealing sacral pelvic dissociation evident by the widening of the sacroiliac (SI) joints bilaterally. Sagittal (right) CT image revealing the widening of the L5-S1 disc space with posterior listhesis and widening of the L5-S1 facet joints with a fracture facet (not seen on the image).

**Figure 2:**
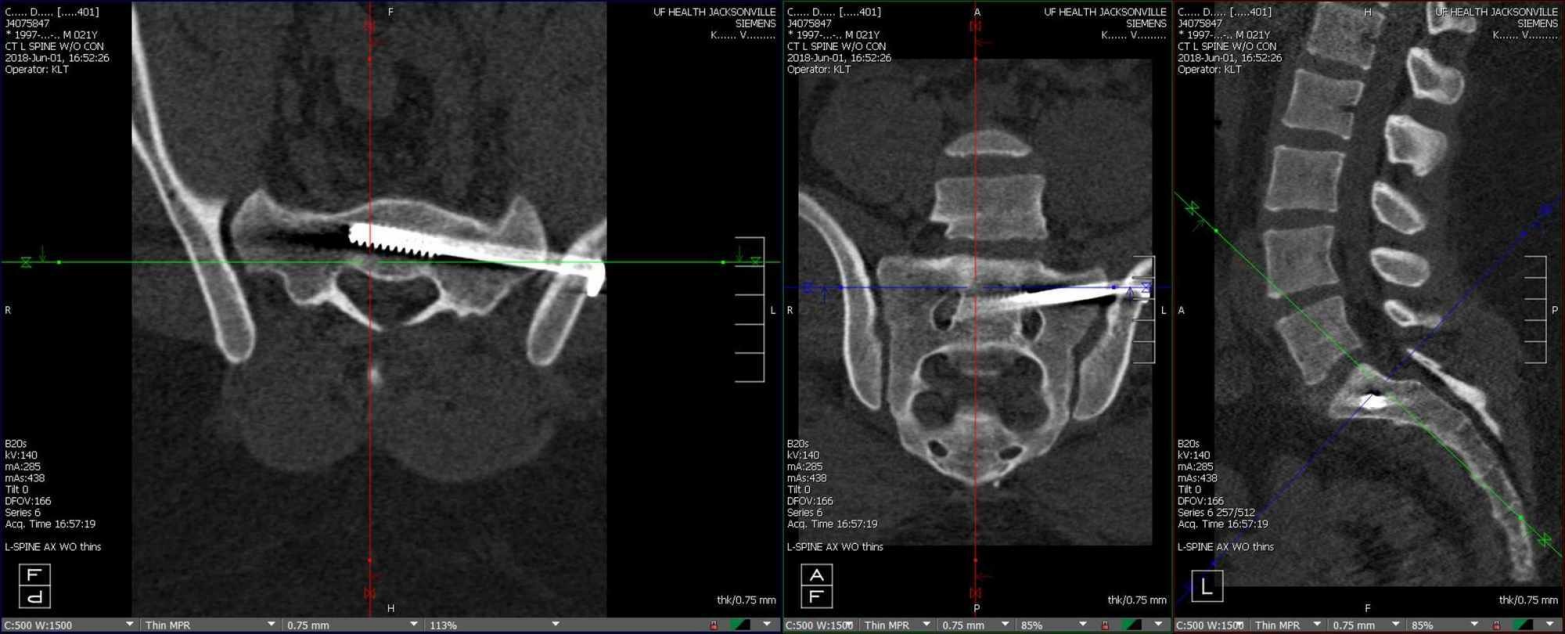
Post sacroiliac stabilization Axial (left), coronal (center), and sagittal (right) CT images revealing post insertion of the left sacroiliac (SI) screw. The SI screw appears to occupy the sacral vertebral body.

Once clinically stabilized, the initial musculoskeletal examination revealed severe left hip, right upper extremity and right lower extremity pain with a deformity of the lower extremity along with bilateral lower extremity (BLE) weakness and inability to move them. Computed tomography (CT) imaging of the pelvis and femur indicated a diastasis of the left sacroiliac joint and a displaced mid-diaphysis fracture of the right femur with lumbar L5-sacral pelvic dissociation (Figure 1). Further imaging with a computed tomography angiography (CTA) scan of the chest revealed a posttraumatic aortic injury of the posterior aortic arch in the region of the ligamentum arteriosum with a focal pseudoaneurysm formation. He underwent an emergent thoracic endovascular aortic repair (TEVAR) to secure his grade 3 blunt thoracic aortic injury (BTAI), followed by emergent intramedullary nailing of the right femur with internal fixation of the left sacroiliac joint using a single sacroiliac screw (Figure 2).

Once systemically stabilized, a detailed neurological assessment revealed a left monoplegia with sensory loss in the left lower extremity up to the T12/L1 region, as well as right lower extremity weakness and patchy sensory loss less than the left side with bladder and bowel involvement, clinically a cauda equina injury worse on the left than the right side. A CT scan of the lumbar spine revealed a right S1 superior articular process fracture with associated subluxation of the bilateral L5-S1 facet joints, vertically distracted L5 vertebra from S1 and anterior subluxed with a nondisplaced L5 spinal process fracture, and bilateral sacroiliac joint subluxation, left worse than right with a left SI screw already inserted for stabilization.

Although the initial plan was to perform an open reduction and internal fixation via the posterior approach, the large, left sacral-pelvic screw appeared to occupy the entire sacral vertebral body, making a sacral screw insertion challenging. We proceeded with a staged 360 procedure, an anterior lumbar interbody fusion at L5-S1 followed by a posterior lumbar fusion across L5-S1.

Surgical caveats

**Figure 3:**
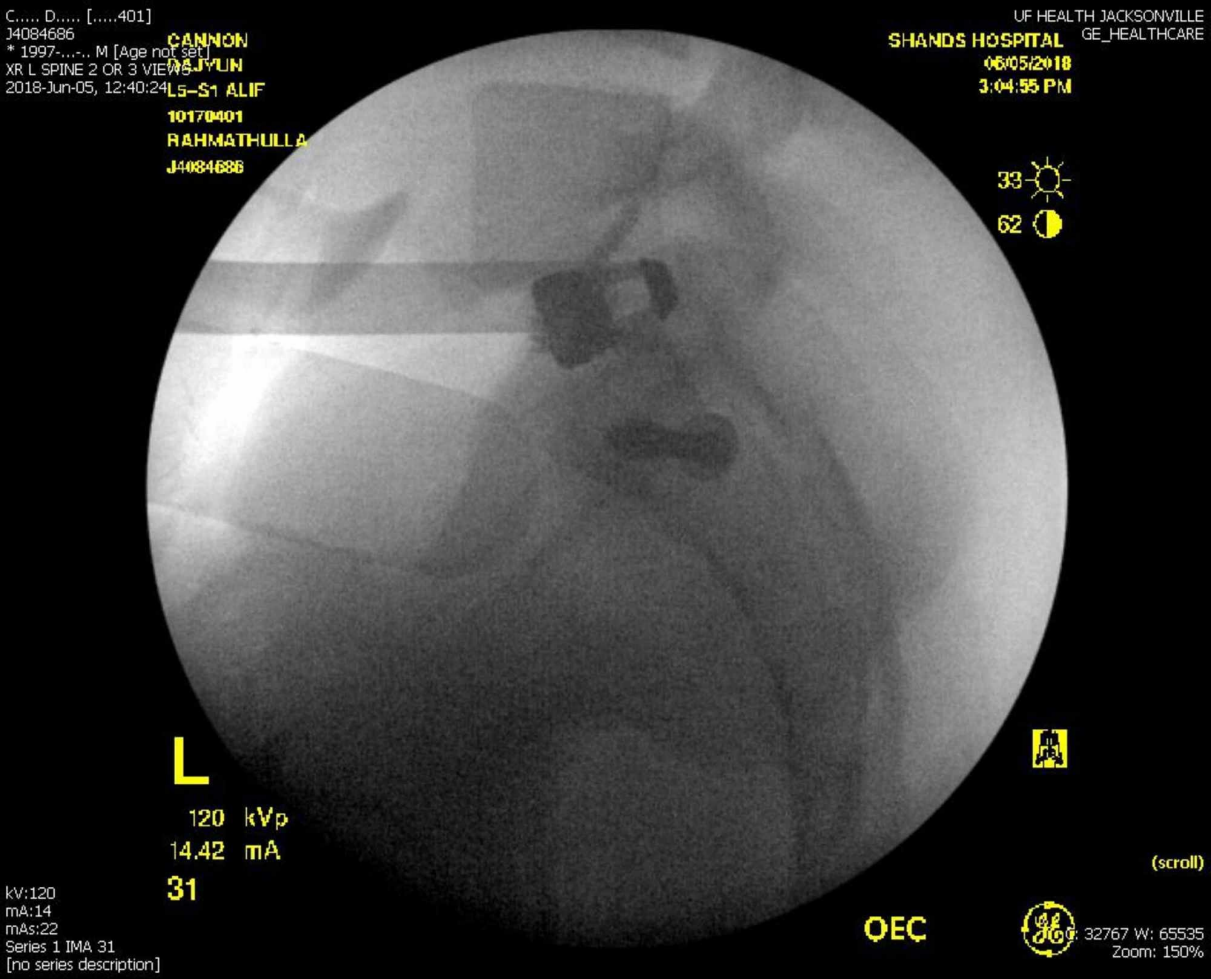
Intraoperative lateral X-ray following the anterior lumbar interbody fusion Intraoperative lateral X-ray view of the L5-S1 region revealing the anterior interbody cage with screws; the largest cage size was inserted, which was marginally displaced up to the posterior vertebral body edge, with the screws getting good purchase without the cage going into the spinal canal.

In a traumatic TLSD, requiring a 360 procedure, it is preferable to be prepared with an anterior lumbar plating system and the anterior interbody cage with a plate rather than a standalone implant. Due to the complete ligamentous disruption, appropriate sizing and cage insertion is a challenge; in our case, we used a standalone anterior lumbar inter-body fusion (ALIF) cage with integrated screws and the vertebra tended to slip away while inserting the screws with a tendency for the graft to slip posteriorly. The easy distractibility can make the operating surgeon increase the size of the interbody implant and the surgeon has to decide the best graft size (Figure 3). This means the safest and most stable graft in the circumstance. In our case, we used a standalone implant requiring us to upsize the graft to get a good fit. Additionally, the standalone ALIF cage has a tendency to slip posteriorly during the insertion of the fixation screws. The screws need to obtain purchase within the endplates and, due to the extensive nature of ligamentous disruption and easy distractability, tend to skive, resulting in the graft moving posteriorly. ALIF cage retrieval may be challenging in situations of posterior migration. Hence, we recommend that surgeons keep a backup option available to them, using alternatively an interbody ALIF spacer with a standalone plate or buttress screw to stabilize the dissociated segment in TLSD once the cage is appropriately inserted.

**Figure 4:**
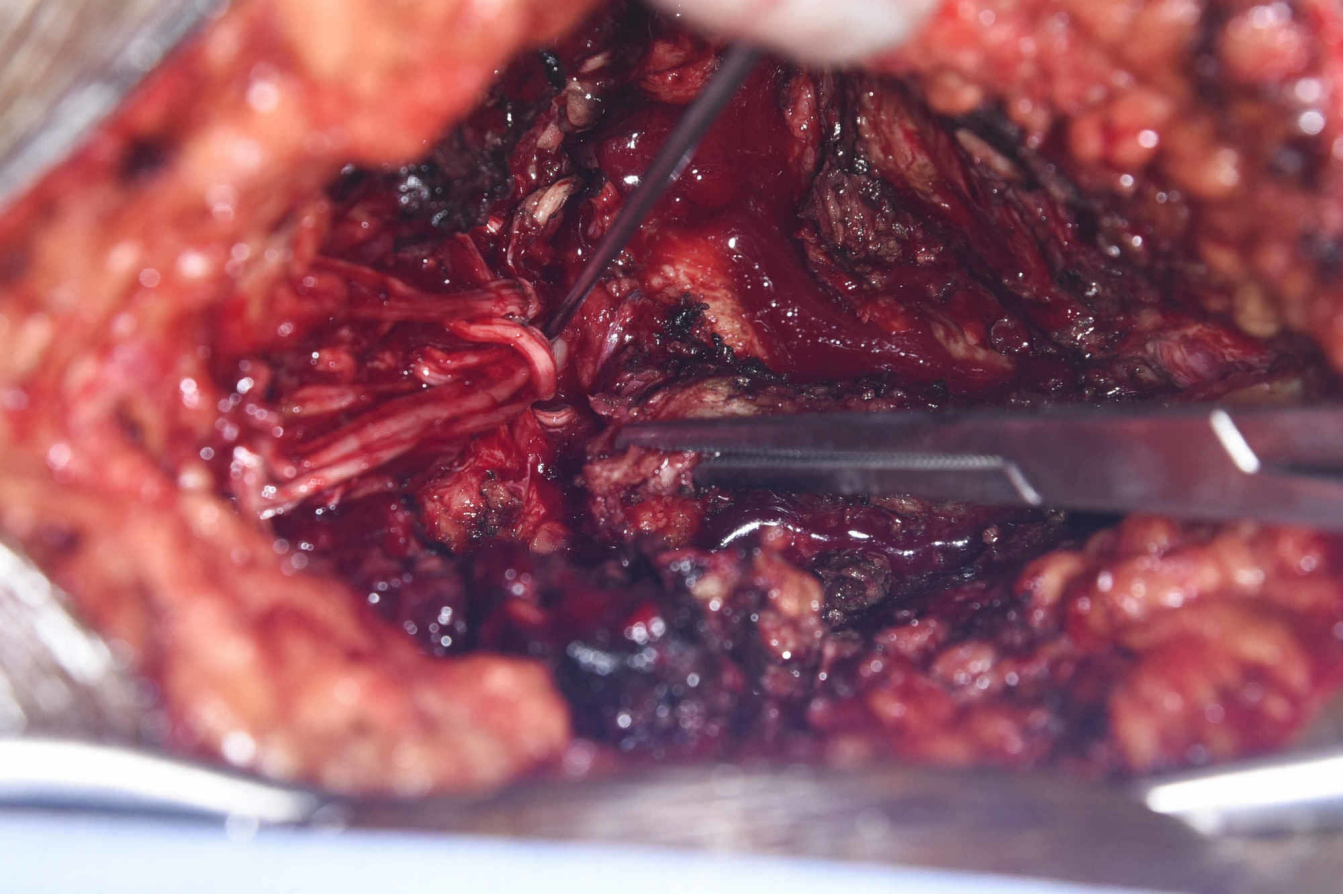
Posterior exposure revealing the avulsed cauda equina and L5-S1 defect The dura was torn completely and the cauda equina was avulsed and could be seen emerging from the L5-S1 defect with few filaments in the paraspinal space. The Kocher clamp is on the L5 spinous process and the dissector is within the avulsed nerve root filaments.

**Figure 5:**
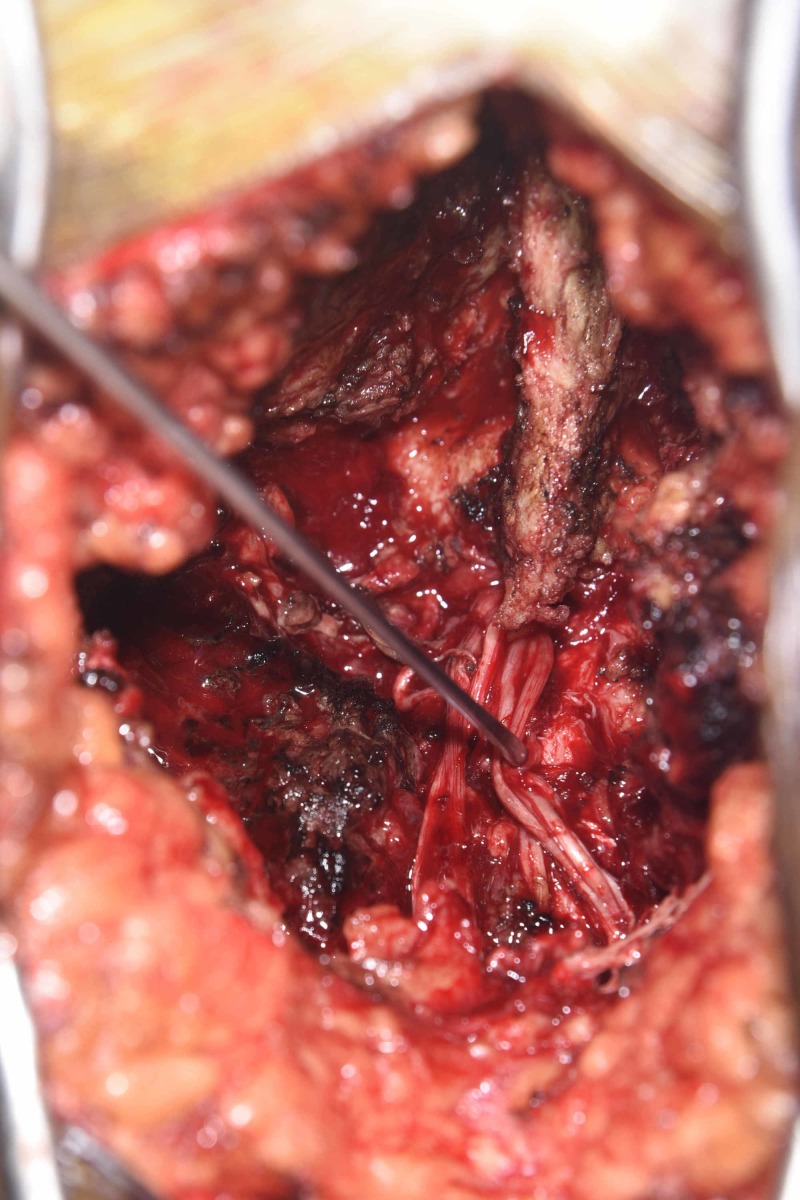
L5-S1 defect with open dura The spinous process of L5 can be seen superiorly with the nerve hook under the avulsed roots in the distracted space between L5 and S1. The nerve roots were gently re-inserted within the canal and duraplasty with Duragen, fibrin glue was performed in an onlay manner, tacking the edge of the Duragen to the adjacent dura and covering the defect. Duragen: Integra LifeSciences, New Jersey

**Figure 6:**
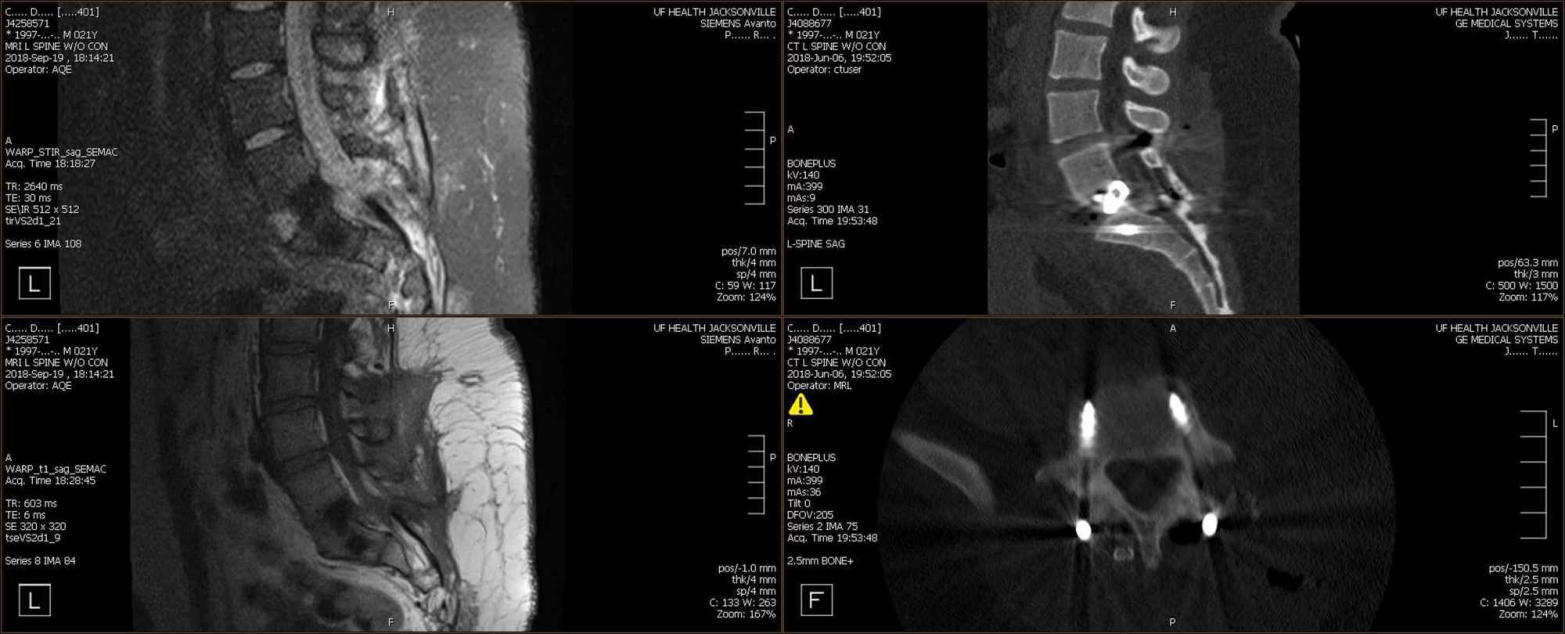
Postoperative follow-up magnetic resonance imaging (MRI - left upper and lower) and computed tomography images (CT - right upper and lower quadrants) MRI follow-up at three months, with the canal widely decompressed and an evident defect at L5-S1 without any evidence of a pseudomeningocele. CT scans revealed anterior and posterior implants in place.

**Figure 7:**
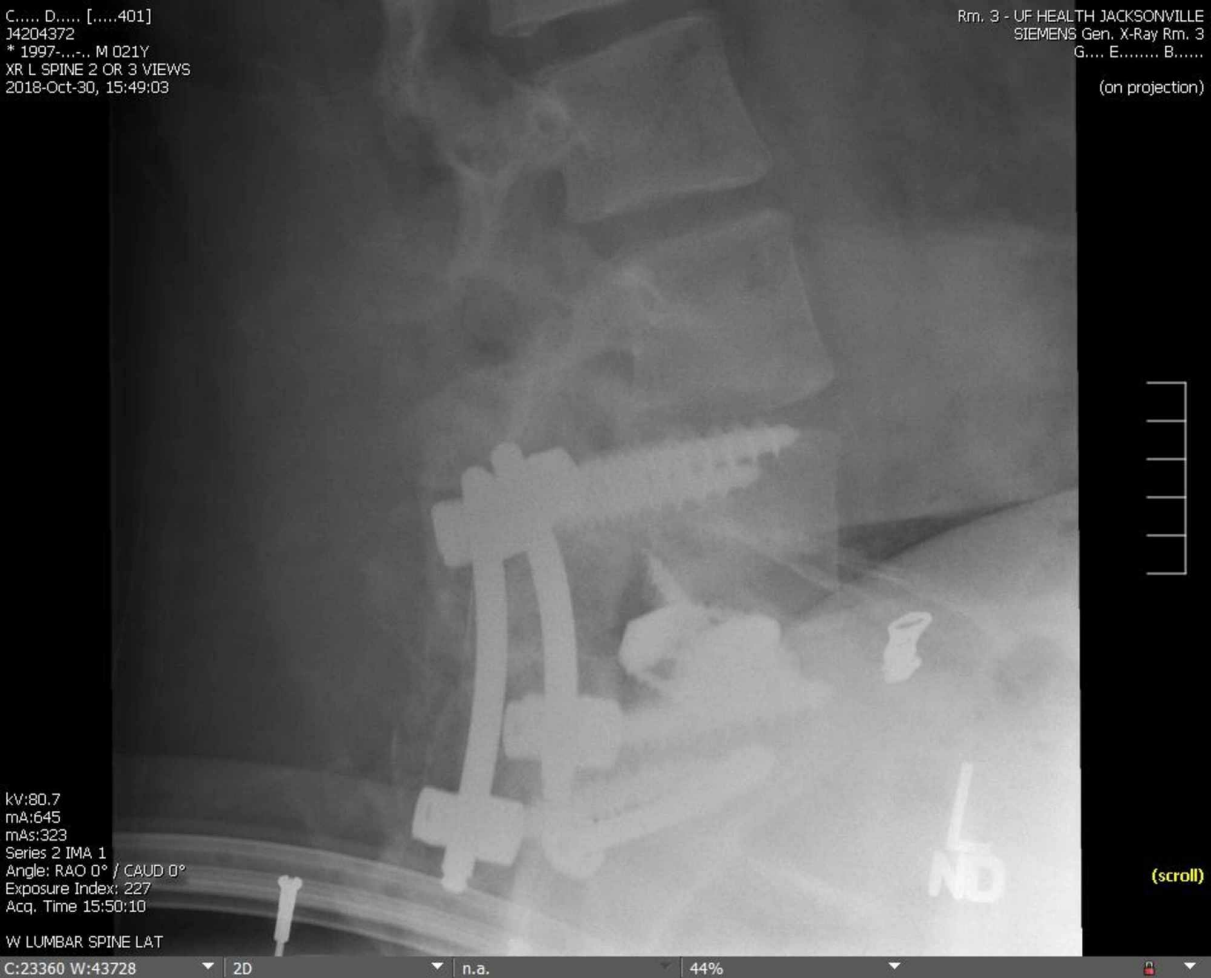
Lateral L-S X-ray at six months Postoperative X-ray revealing the anterior and posterior implant in a good position at six months

Following the anterior procedure, we felt a short segment posterior arthrodesis across L5-S1 would be adequate to stabilize the L-S dissociation and proceeded with a posterior lumbar fusion. Both procedural techniques have been extensively published in the literature, with a range and variety of implants and instruments that can be utilized [6-9]. With the posterior procedure, the ligamentous complex was disrupted and there was a large gap in the lamina of L5-S1. The dura was torn completely, and the cauda equina was avulsed and could be seen emerging from the L5-S1 defect with few filaments in the paraspinal space (Figures 4-5). On electromyography (EMG) testing, there was no electrical activity on many of the nerve roots. These were gently reinserted into the open dural tube and duraplasty performed with an artificial dural substitute and fibrin glue along with gelfoam and some blood to form a watertight closure. The pedicle screw purchase posteriorly was excellent, and the L5-S1 segment appeared stable. There was no evident injury above the L5-S1 level and, hence, we decided not to extend the procedure above (Figures 6-7). At one year, the patient has motor and sensory function in the right lower extremity, with left monoplegia and continued bowel/bladder dysfunction at baseline.

## Discussion

Traumatic lumbosacral dissociation is an unusual spondylolisthesis involving L5-S1 that is estimated to account for approximately 1% of total spinal fractures [5]. It is typically the sequelae of a high-energy distraction injury, such as a motor vehicle collision, most likely involving healthy adults. Because of the high-energy nature of the injury, several concurrent organ system damages occur, including to the musculoskeletal and neurovascular elements, which may occur local or distal to the principal site. Although TLSD incidence is small, there are numerous case reports and small case series documenting the unique perspectives of this fracture-dislocation, dating back to the mid-20th century when Watson-Jones first described it [10]. However, as shown by our representative literature search on TLSD, the classification and subsequent medical management of TLSD remains a controversial topic, with high variability, and the technique and approach commonly depending on surgeon preference. TLSD typically results in significant long-term neurological dysfunction and disability. Adelved et al. reported that among 13 patients with TLSD who were followed over an average period of 7.7 years, while the majority were able to perform the activities of daily living (85%), many exhibited reduced quality of life in measures such as neurological deficits (85%), sexual dysfunction (62%), and altered bladder function (69%) [11].

In our case, we highlighted the complex etiology and unique management of LSD. Our patient is an otherwise healthy, morbidly obese young male who presented to the emergency room after a high-energy trauma with lumbar spinal cord injury, with symptomatology emblematic of cauda equina syndrome, additional to injuries to his vasculature and musculoskeletal elements. Appropriate identification of this injury requires careful evaluation on admission, utilizing advanced trauma life support (ATLS) guidelines with an appreciation of the variability in clinical manifestations and associated neurological/musculoskeletal damage. The sequential evaluation and use of multi-modality imaging modalities help diagnose these complex injuries [5]. Recent literature reports that a majority of these injuries result in an anterior dissociation of the lumbosacral joint, with posterior dissociation and lateral dissociation being among the least common [4,12-15]. The management of TLSD is typically a complex and multifactorial effort because of the multisystem involvement including injury to major vessels and abdominal and spinal injury. TLSD requires the stabilization of concomitant injuries and patient-specific evaluation of spinal dissociation severity. Due to the complete osseoligamentous complex disruption, surgical stabilization with possible decompression is almost universally required, unless patients are critically injured and unlikely to survive their injury [4-5,16]. The most common approaches used involve anterior lumbar inter-body fusion (ALIF), a posterior lumbar interbody fusion (PLIF), a combined approach using PLIF and transforaminal lumbar interbody fusion (TLIF), circumferential lumbar fusion (CF), or newer and increasingly more popular minimally invasive techniques. The optimal surgical technique varies depending on the extent of the injury, the clinical condition of the patient, and the association of other systemic injuries.

Current literature on classification and treatment algorithms for high-energy lumbosacral dissociations and fractures is suboptimal, as they are primarily descriptive and lack prognostic value [2]. However, there is a consensus that treatment ordinarily involves surgical decompression and there are several techniques to choose from, which are based on published studies that provide low levels of evidence (level IV) [4-5,16]. Lehman et al. provided a novel classification system consisting of thresholds classifying surgical and nonsurgical candidates, determining the optimum operative technique by generating a composite injury severity score based on a weighted score from categories such as injury morphology, posterior ligamentous complex integrity, and neurological status [2]. Although further longitudinal studies are required in order to assess the effectiveness of this classification system, it is a step in the direction towards a standardized classification system for TLSD.

Our patients' coordinated and sequential care by the orthopedic, vascular, neurosurgery, and surgical intensive care teams, as his surgical management progressed, features the multidisciplinary effort and need for multispecialty team care required to treat TLSD in a timely and effective manner. The unpredictable nature of TLSD injuries dictates a combination of procedural and medical management flexibility in order to provide greater stability and improved patient outcomes.

Outcomes based on our literature review

Table 1 lists the outcomes of various procedures based on our literature review.

**Table 1:** Management and outcomes of traumatic lumbosacral dissociation (TLSD) TLSD = traumatic lumbosacral dissociation. LSD = lumbosacral dissociation. * Only represents number of patients treated operatively; total cohort consisted of 13 patients, eight of which were managed medically. a Represents longest follow-up time only.

Published literature	Year	N	Injury	Procedure(s)	Follow-up^a^	Outcomes
Murata [17]	1999	1	Horizontal basal fracture of L5 lamina w/rotational instability	(1) Posterior open reduction, (2) modified laminaplasty of L4, (3) posterior-lateral arthrodesis of L5-S1 w/pedicle-screw instrumentation	1.5 years	Semi-independent ambulation
Mukundala [12]	2001	1	Comminuted burst fracture and posterior dislocation of L5 onto S1	(1) Open reduction, (2) Posterior fusion w/Hartshill rectangle	-	-
Cruz-Conde [6]	2003	1	Anterior lumbosacral dislocation	(1) Open posterior reduction fixation, (2) fusion w/pedicle screws	5 years	Asymptomatic
Vialle [14-15]	2005	1	Lateral dislocation of lumbosacral junction w/o anterolisthesis of L5	(1) Posterior open reduction, (2) L5-S1 stabilization with Tenor instrumentation	6 years	Asymptomatic
Lu [4]	2009	1	Complete anterior lumbosacral spondyloptosis	(1) L4/L5 surgical reduction, (2) posterior-lateral spinal fusion w/pedicle screw instrumentation, (3) sacropelvic fixation	1.4 years	Semi-independent ambulation
Angthong [18]	2010	1	Grade II spondylolisthesis of L5-S1	(1) Posterior decompression, (2) reduction, (3) stabilization, (4) fusion w/instrumentation	1 year	Partial neurological recovery
Helgeson [19]	2011	23	Combat-related lumbosacral dissociations with zone III sacral fractures	No fixation (9), sacroiliac screw fixation (8), posterior spinal fusion (5) and sacral plate (1).	1 year	operative stabilization promoted early mobilization, high post-operative infection risk
Kang [1]	2012	20	Combat-related lumbosacral dissociation with sacral and lumbar fractures	Posterior spinal fusion (12, 60%), sacroiliac screw fixation (7, 35%), and combined anterior-posterior fusion for associated L3 burst fracture (1, 5%)	Mean 85.9 months (range: 39.7-140.8 months)	(4) postoperative wound infection; (2) underwent re-operation. (17) no longer on active duty military service. (8) persistent bowel dysfunction (9) persistent bladder dysfunction. (15) chronic low back pain. (17) ambulating (5) documentation of running following surgery.
Grivas [9]	2012	1	Unilateral L5-S1 dislocation w/posterior ligamentous complex disruption	(1) Open reduction, (2) posterior instrumentation system	6 years	Asymptomatic
Gabel [8]	2015	1	L5-S1 fracture-dislocation with retrolisthesis of L5 over S1	(1) L2-sacroiliac joint posterior instrumented fusion, (2) L5 vertebrectomy	-	-
Robbins [16]	2015	2	(1) L5-S1 facet joint fracture with grade II anterolisthesis, (2)	(1) open reduction internal fixation	Post-operatively	½ asymptomatic, ½ neurological dysfunction
Yazdi [20]	2015	1	L4-S1 spondylolisthesis	(1) spinopelvic fusion w/instrumentation	2.6 years	Asymptomatic
Arandi [13]	2015	1	Right lateral fracture-dislocation at L5/S1 with zone 2 right sacral fracture	(1) Combined anterior and posterior arthrodesis, (2) posterior decompression w/instrumentation	1 year	Semi-independent ambulation
Formby [7]	2016	20	Combat-related LSD	(1) posterior spinal fusion, (2) sacroiliac screw fixation, (3) combined anterior-posterior fusion	7.2 years	Most with neurological, bladder, and pain dysfunction
Adelved [11]	2016	5*	Zone III fracture (region of the central sacral canal) (Denis)	(1) Open reduction, (2) internal fixation w/iliolumbar interpedicular screws, (3) concomitant sacral laminectomy	7.7 years	Most with neurological dysfunction

## Conclusions

We present an uncommon case of traumatic lumbosacral dissociation, and our report describes a short segment stabilization strategy. TLSD requires individually tailored approaches and the number of levels and types of procedures depends on the patient, injury, and other co-morbid factors. Although we do not recommend short segment stabilization in these cases, in certain clinical situations, this may be warranted.
